# Comparing the CO-OP Approach^TM^ to Usual Occupational Therapy for Adults with Executive Dysfunction Following Acquired Brain Injury: A Randomized Controlled Trial †

**DOI:** 10.3390/brainsci15111195

**Published:** 2025-11-05

**Authors:** Deirdre R. Dawson, Nicole D. Anderson, Yael Bar, Malcolm A. Binns, Adora Chui, Anne W. Hunt, Emily Nalder, Merrick Zwarenstein

**Affiliations:** 1 Department of Occupational Science & Occupational Therapy & Rehabilitation Sciences Institute, University of Toronto, 500 University Avenue, Toronto, ON M5G 1V7, Canada; anne.hunt@utoronto.ca (A.W.H.); emily.nalder@utoronto.ca (E.N.); 2 Rotman Research Institute, Baycrest Academy for Research and Education, 3560 Bathurst Street, Toronto, ON M6A 2E1, Canada; nanderson@research.baycrest.org (N.D.A.); ybar2@baycrest.org (Y.B.); mbinns@research.baycrest.org (M.A.B.); 3 Departments of Psychology and Psychiatry, University of Toronto, 100 St. George St., Toronto, ON M5S 3G3, Canada; 4 Division of Biostatistics, Dalla Lana School of Public Health, University of Toronto, 155 College Street, Toronto, ON M5T 3M7, Canada; 5 Institute of Health Policy, Management and Evaluation, University of Toronto, 155 College Street, Toronto, ON M5T 3M6, Canada; adora.chui@utoronto.ca; 6 Department of Family Medicine, Epidemiology & Biostatistics, Schulich Medicine & Dentistry, Western University, London, ON N6A 3K7, Canada; mzwarens@uwo.ca

**Keywords:** executive dysfunction, rehabilitation, strategy-training, stroke, traumatic brain injury, cognitive orientation to daily occupational performance, occupational therapy

## Abstract

Impairments of executive function following acquired brain injury including stroke (ABI) contribute significantly to long-lasting everyday difficulties in life. Pilot work on the CO-OP Approach^TM^ (Cognitive Orientation to daily Occupational Performance Approach), a contextualized strategy training intervention, with ABI adults with executive dysfunction showed improved performance on untrained everyday life tasks and cognitive flexibility. **Objective:** Our objective was to determine the efficacy of the CO-OP Approach relative to usual occupational therapy (UOT) for community-dwelling adult survivors of ABI with executive dysfunction. **Methods:** Eighty-seven participants were randomized to receive CO-OP (*n* = 45) or UOT (*n* = 42) in their homes. All participants identified five personally meaningful, everyday life goals (using the Canadian Occupational Performance Measure (COPM)) and received up to 15 one-hour treatment sessions twice per week. Three goals were trained, and two were untrained. Interventions were provided by occupational therapists registered with their regulatory college. The CO-OP group was trained to apply a meta-cognitive strategy to three goals. The UOT group received therapy based on the clinicians’ (experienced in community settings) determination. Testers were masked to the participants’ group. Analysis at the primary outcome (Post-test, ~10 weeks following baseline) was on an intent-to-treat basis. **Results:** Participants in the CO-OP group had a mean age of 57.5 years, a mean time post-ABI of 5.3 years, and were 57.8% men. Those in the usual OT group had a mean age of 54.7 years, a mean time post-ABI of 6.2 years, and were 69.0% men. The CO-OP group reported statistically significant higher improvements on COPM performance and satisfaction scores post-test for untrained and trained goals. However, these benefits were not retained at follow-up (three months post-baseline). **Conclusions:** The CO-OP Approach^TM^ shows promise for improving performance in everyday life goals for individuals with chronic ABI relative to usual community occupational therapy. Achieving retention of these benefits remains a challenge.

## 1. Introduction

Many survivors of acquired brain injury (ABI: stroke, traumatic brain injury, and other forms of suddenly acquired brain injury) report extensive and long-lasting disablement, particularly in relation to cognitive skills and activities that demand these skills [1,2,3,4]. Importantly, this disablement is not static. Rehabilitation studies in various domains (motor, cognitive, mood, etc.) have shown that there is a benefit to survivors in the chronic stages of ABI [5,6,7]. Evidence also points to the risk of deterioration after cessation of rehabilitation [8,9] and to cortical degeneration following traumatic brain injury [10,11]. All of this suggests that ABI should be recognized and managed as a chronic health condition and that investigations should be undertaken into the nature and extent of rehabilitation that would provide the most benefit in the chronic stages of ABI [10]. Given that the definitive goal of rehabilitation is individually meaningful participation in everyday life, we believe this should be the primary outcome for investigations.

One factor associated with long-lasting disablement for survivors of ABI appears to be impairments in executive processes, including problems in self-regulation [12]. Executive dysfunction is endemic among survivors of ABI [11] and arguably has the most devastating effects on everyday life, as it is “at the heart of all socially useful, personally enhancing, constructive and creative activities” [13] (p. 281). As executive processes are necessary for conceptualizing, planning, carrying out, and monitoring complex and novel activities of daily living, there is a wide range of difficulties arising from impairments in these processes. At the time the current trial started, meta-cognitive strategy training had been identified as a practice standard for deficits in executive function following TBI [14]. A 2014 systematic review came to the same conclusion, emphasizing the importance of focusing strategy training on everyday problems and functional outcomes [15]. Nevertheless, achieving generalization and transfer of training effects to everyday function continues to be identified as an important challenge to be addressed and measured [16,17,18].

While most health care systems do not provide rehabilitation services in the chronic stages of ABI, in some instances people with ABI are able to receive longer-term services; for example, they can receive these services through privately funded rehabilitation, drop-in centres, and/or by having experienced a significant deterioration in health status that brings them back into the health care system [19]. Thus, an important question related to the nature of services for chronic ABI is whether the intervention being examined provides any benefit beyond that already provided within the boundaries of the health care system. To date, relatively few studies have examined the benefits of a formal strategy training program relative to the benefits achieved with more conventional therapy [20,21]. Using conventional therapy as a control condition allows consideration of whether any of the benefits seen are specific to the strategy training or simply due to added therapy time.

The Cognitive Orientation to daily Occupational Performance (CO-OP) Approach^TM^ is a meta-cognitive strategy training approach appropriate for use with children and adults that, to date, has achieved international recognition (https://www.icancoop.org/ (accessed 16 April 2023)), and has been the focus of two texts [22,23] and five or more review papers [24,25,26,27,28]. This approach has the overarching aims of task acquisition, strategy use, and the generalization and transfer of strategy use as applied to meaningful activities in everyday life [22]. Achievement of these aims has been reported in a series of trials and controlled studies of adults with cognitive impairment arising from ABI, including [6,29,30,31,32]. However, to date, no one has investigated the benefits in a fully powered study in a group of adults with ABI in the chronic stage and determined whether the benefits exceed those of conventional therapy at this stage. The CO-OP Approach for use with adults with ABI is described in detail elsewhere [23]. The combination of its essential elements makes it unique from other meta-cognitive strategy training approaches in several key ways. The training is contextualized by having recipients identify everyday life activities that they need to, and/or want to, perform but with which they are having difficulty. This allows for personally meaningful goals to provide the focus for therapy. Despite substantial evidence that this is an essential part of OT practice, a recent systematic review found that its inclusion is highly variable [33]. The clinician facilitates the therapy using “guided discovery” in order for the recipient to discover and learn specific approaches towards improving performance of their everyday life activities [34]. The clinician and client use a meta-cognitive strategy (Goal-Plan-Do-Check) to structure the process of guided discovery and together analyze where performance is breaking down, and to figure out specific strategies to address these breakdowns. While developed and often used within the context of occupational therapy practice, CO-OP has also been used by other health professionals, including physiotherapists and dietitians [35,36].

The purpose of this study was to examine the benefits that might accrue to individuals with ABI in the chronic stages who received CO-OP. The specific objectives were to discover whether the CO-OP Approach was more effective than conventional occupational therapy for the following:

1. Improving performance of, and satisfaction with, everyday life activities:
  - Activities that were untrained in therapy immediately following intervention (primary outcome, primary endpoint);
  - Activities that were trained in therapy immediately following intervention;
  - Activities that were trained and untrained at three-month follow-up (secondary endpoint).
2. Achieving overall improved executive function and overall improved community integration (secondary outcomes).

## 2. Methods

### 2.1. Design

This study was a randomized controlled trial with a three-month follow-up. The trained, independent outcome assessors were masked. Participants with a history of ABI were randomly allocated to receive therapy using CO-OP or to receive usual occupational therapy (UOT). Participants were aware that they would be randomized to one of two treatment protocols but were not told which therapy they would receive. All participants were allotted up to 15 h of therapy (2, 1 h sessions twice/week). Post-testing took place at the end of the therapy (about a 10-week interval) and follow-up testing took place three months later. Further details of the design and the entire study protocol are available elsewhere [37]. The study was approved by the Baycrest Research Ethics Board and registered at clinicaltrials.gov (Identifier: NCT01414348). All participants provided informed, written consent. A subset of the results was reported in a book chapter [38].

### 2.2. Recruitment Procedures and Randomization

Community-dwelling participants were recruited via (a) an institutional research subject pool at Baycrest Health Sciences (a hospital fully affiliated with the University of Toronto), (b) advertisements distributed throughout a network of community agencies providing services to survivors of ABI within the Greater Toronto Area, and (c) recruitment talks at various ABI survivor groups within the Greater Toronto Area. Participants were screened by telephone to determine initial eligibility using the following criteria: (a) 18 years of age or older; (b) living within a 100 km radius of Baycrest Health Sciences; (c) able to communicate in the English language with or without communication aids, including hearing aides; (d) diagnosis of an ABI six or more months previously (including stroke, traumatic brain injury, anoxia, meningitis, subarachnoid hemorrhage, and non-malignant tumour removal unrelated to a congenital, developmental, or degenerative condition); (e) failure of one or more of the first five tasks from the EXIT-25 (tasks amenable to administration via telephone) [39]; (f) absence of report of other significant neurological or psychiatric history (e.g., multiple sclerosis or psychiatric illness requiring hospitalization); (g) absence of concurrent moderate or severe depression as measured on the Center for Epidemiological Studies Depression Scale (score of ≥26) [40]; (h) absence of self-report of substance misuse; (i) not receiving other occupational therapy services; and (j) able to identify four or more everyday activities with which they were having difficulty and wanted or needed to improve.

Based on data from our previous studies [5,6], we estimated that a sample of 45 per group was needed to detect a moderate effect size (Cohen’s *d* ≥ 0.5) with 80% power and an alpha of 5%. We planned to oversample by 10%, aiming to recruit 50 people/arm.

Following baseline testing, eligible and consenting participants were randomized to receive either CO-OP or UOT. Randomization was performed in blocks of four using a random numbers table generated by the statistician (MB). An administrative assistant not associated with the study prepared numbered sealed envelopes so that the principal investigator (PI), study coordinator, research assistants (RAs), and treating therapists remained masked to the randomization throughout the study. Research assistants and treating therapists were also masked to the study objectives. Research assistants were masked to therapy protocols, and treating therapists were aware only of the protocol for the therapy they were providing (either CO-OP or UOT). Following baseline testing and a review of inclusion criteria and participant registration, the study coordinator opened the next sealed, opaque envelope in sequence to allocate the next participant. The PI, RAs, and statistician remained masked to allocation.

### 2.3. Assessment and Intervention Procedures

All testing (baseline, post-intervention, and three-month follow-up) was performed at Baycrest. Trained and reliable assessors, masked to group allocation and intervention content throughout the study, administered a set of neuropsychological assessments, a questionnaire on executive function, and a questionnaire on community integration at each time point. Following these tests, trained research therapists (also masked to group allocation) who were not treating therapists administered the primary outcome measure, the Canadian Occupational Performance Measure (COPM), a standardized, semi-structured interview [41].

All treating therapists were occupational therapists registered with the regulatory body, the College of Occupational Therapists of Ontario. Those providing CO-OP were trained by a certified CO-OP instructor (DD). Those providing UOT had experience working with adults with ABI and were supervised by a highly experienced neurorehabilitation OT working as a team leader in a rehabilitation setting that provided inpatient and outpatient therapy for people with ABI. Treating therapists and participants in both the CO-OP and UOT groups received the list of goals and were aware of which goals were for training and which goals were not to be discussed or practiced in therapy sessions (i.e., untrained goals). They were also provided with participant contact information and the diagnosis. Therapy was provided in the participants’ home environments.

Treatment fidelity in both groups was facilitated by regular meetings with the CO-OP instructor for therapists administering the experimental protocol and by the OT team leader for those administering UOT. All therapists were provided with a template for documentation of observations (e.g., persons present, adverse events, and changes in routines) and for a brief description of what was performed at the therapy session. These were used as the basis for the meetings with the CO-OP instructor and OT team leader. Treating therapists for both groups were masked to the intervention provided in the other treatment arm, as was the supervisor of the UOT group. Treating therapists were not provided with the study protocol.

### 2.4. Experimental Intervention—Adapted CO-OP Approach

Essential elements of the CO-OP include the following [42]: (1) Self-selection of personally meaningful goals aimed to elicit greater participant engagement in the treatment process. This element was also part of UOT, although we are aware that this does not always occur in clinical practice [33]. (2) Application of a meta-cognitive strategy (Goal-Plan-Do-Check) for participants to support goal attainment. Participants were taught to use the strategy iteratively to work toward their goals by generating, selecting, executing, and then checking or evaluating the results as a way of problem-solving. Workbooks were provided to participants to facilitate their own tracking of their progress towards achieving their goals. (3) Use of guided discovery techniques by therapists to endorse participant skill development, to identify performance issues (i.e., dynamic performance analysis), and to originate plans to address these issues. Through the use of guided discovery, therapists may ask questions and provide observations and feedback as a way of drawing participants’ attention to performance breakdown and feasible plans to address this breakdown, without telling participants what to do [36]. Rather, participants are guided to decide which plans they will implement, execute, and self-monitor. (4) Focus on generalization and transfer of learning through engaging participants in thinking about and discussing how else they have applied the identified strategies, or how they may continue to use them beyond the initial opportunity. For this study, the protocol allowed for up to 15 individual training sessions of up to 60 min each, twice/week. A session guide was developed by the authors (DD, AH) and provided to the treating therapists.

### 2.5. Control Intervention—Usual Occupational Therapy (UOT)

As treating therapists in the UOT arm were clinicians experienced in working with clients with ABI in the public and private sector in rehabilitation and/or community settings, they delivered this arm of the study by drawing on their clinical expertise. Treatment encompassed specific training on functional activities, recommendations for task modification and/or environmental adaptation, and recommendations and/or provision of assistive equipment. This intervention was considered comparable to what might be provided by a community occupational therapist, apart from having participants set personally meaningful goals through the COPM interview.

The supervisor of the UOT therapists was not made aware of the experimental protocol but was asked to defer, in supervisory meetings, from suggesting using any form of strategy training and to monitor supervision sessions for mentions of the use of strategy training. The supervising therapist was asked to instruct therapists to choose another approach, although, notably, the suggested use of strategy training never occurred. Therapists delivering UOT were also instructed to use up to 15 individual training sessions of up to 60 min each, twice/week. In Ontario, Canada, dosage of community-based therapy varies considerably. Thus, an upper cap of 15 h was set to align with the CO-OP protocol. Therapy was discontinued in the UOT group in collaborative decision-making between the therapist and participant. No directive was given to treating therapists as to how or when to end therapy, except that a maximum of 15 h was permitted.

### 2.6. Measures

Participants were characterized during in-person baseline testing using socio-demographic (age, gender, education, estimated pre-morbid IQ, living arrangement, employment status, and depression symptoms) and time post-ABI variables. Baseline self-care status was derived from the Mayo Portland Adaptability Inventory described below [43]. Pre-morbid IQ was estimated using the Shipley Institute of Living Scale: Vocabulary Subtest [44]. Depression symptoms were assessed using the Patient Health Questionnaire-9 (scores of ≤9 indicate no or mild depressive symptoms) [45]. To determine if participants’ engagement with the received intervention varied between the two arms, the Hopkins Rehabilitation Engagement Rating Scale (54 items) was used post-intervention [46].

### 2.7. Primary Outcome Measure

The primary outcome measure was the Canadian Occupational Performance Measure (COPM), a standardized, semi-structured interview that is widely used in intervention research, including in previous studies on the CO-OP approach [47,48,49,50]. The COPM has proven to be a valid and reliable measure of change in participants’ self-identified goals [41]. During the COPM interview, participants set personally meaningful, everyday life goals, with which they were experiencing difficulty. Participants then rated these goals on a 10-point Likert-type scale by importance (1 = not important at all to 10 = extremely important), current performance ability (1 = not able to do it at all to 10 = able to do it extremely well), and satisfaction with performance (1 = not satisfied at all to 10 = completely satisfied). The five or six most important goals were selected for study purposes prior to the start of treatment. The most important participant-ranked goal was trained, as were two additional goals that were randomly selected from the other goals. Thus, three goals were trained, and the remaining two or three goals were untrained. Although participants were free to work on their untrained goals independently, no encouragement or discussion of these goals was provided by the treating therapists for either group. However, therapists in both groups were free to work with participants on other issues that came up in relation to the goals being trained.

The primary outcome was the change in participants’ self-rated performance and satisfaction with performance of the untrained goals (an indicator of generalizability) from pre- to post-intervention. As a change of approximately 2 points on the COPM performance and satisfaction scales is considered the minimally clinically important change [51], we also planned to note the magnitude of change of these scores.

### 2.8. Secondary Outcome Measures

Change scores on the COPM from pre-intervention to follow-up testing were examined to determine maintenance of training effects.

To further assess the potential for transfer of training effects, measures of executive function and community integration were used. Executive function processes were measured using self-ordered pointing [52] and two tests from the Delis–Kaplan Executive Function System: Trail Making-Condition 4 and Fluency [53]. The impact of executive dysfunction in everyday life was measured using the 75-item Behavioural Rating Inventory of Executive Function—Adult (BRIEF-A) [54]. We used the Behavioural Regulation Index (BRIEF-BRI) to investigate participants’ ability to regulate and monitor emotional responses and to show flexibility in problem-solving, and the Metacognition Index (BRIEF-MI) to investigate participants’ ability to initiate, plan, and organize approaches to problem-solving. The BRIEF-BRI contains 30 items, and the BRIEF-MI contains 40 items [55]. Each item is scored on a three-point frequency scale; thus, raw scores range from 0 to 90 for the BRIEF-BRI and 0 to 120 for the BRIEF-MI. Lower scores indicate higher levels of behaviour regulation and/or metacognition.

Community integration was measured using the Mayo-Portland Adaptability Inventory: Adjustment and Participation Subscales (MPAI-4), which is widely used in ABI research [43,56]. The Adjustment and Participation subscales each contain eight items with questions on mood, social interactions, work and school, and financial management. Scores range from 0 to 32 for each scale, with lower scores representing better adjustment and participation [43].

### 2.9. Analyses

Data analyses were conducted using R (R Foundation for Statistical Computing, Version 4.5.1) and the IBM SPSS Statistics (2025, Version 31.0). The data were cleaned for accuracy. Descriptive statistics were used to characterize participants’ baseline socio-demographic, event-related, and neuropsychological status. Proportions were reported for categorical variables (i.e., gender, living arrangement, and significant other involvement), and means and standard deviations were calculated for continuous variables. Effect size indices (Cohen’s *d* and *w*, respectively) were also calculated for between-group comparisons. For Cohen’s *d*, effect sizes of about 0.2 are small, 0.5 are medium, and 0.8 or more are large; For Cohen’s *w*, effect sizes of about 0.2 are small, 0.3 are medium, and 0.5 are large [57].

To determine if the CO-OP approach was more efficacious than UOT, analyses on the primary outcome (change in performance from pre- to post-intervention measured using the COPM) at the primary endpoint (post-intervention) were conducted in conformity with the intent-to-treat (ITT) principle, where participants were analyzed within their allocated treatment group regardless of study completion [58]. A multiple imputation technique (mi function, in R) was used to accommodate missing data with minimal bias, relating partially observed performance scores to complete data for age, gender, education, depression symptoms, average pre-intervention performance score, and binary treatment arm. Analyses of covariance models (ANCOVA) were run for COPM scores (performance of untrained goals, satisfaction with untrained goals, performance of trained goals, and satisfaction with trained goals) at the primary endpoint (post-intervention) with pre-intervention ratings entered as a covariate. Baseline variables with between-group effect size differences of at least medium size (Cohen’s *d* = 0.5, Cohen’s *w* = 0.3) were included in models to determine whether they were significantly associated with the treatment effect.

The types of goals set were categorized descriptively as activity and participation goals or body function and structure goals, following the categories of the International Classification of Functioning Disability and Health [59].

Per-protocol analyses were used for COPM outcomes at the secondary time point (3-month follow-up) and secondary outcomes at post-test and 3-month follow-up using repeated measures ANOVA. Effect sizes were calculated for these analyses using partial eta squared (η^2^), for which values range from 0 to 1. Partial eta squared values around 0.01 were considered small, around 0.06 were considered moderate, and greater than or equal to 0.14 were considered large [60].

An alpha level of 5% was set for all hypothesis testing.

## 3. Results

**Figure 1:**
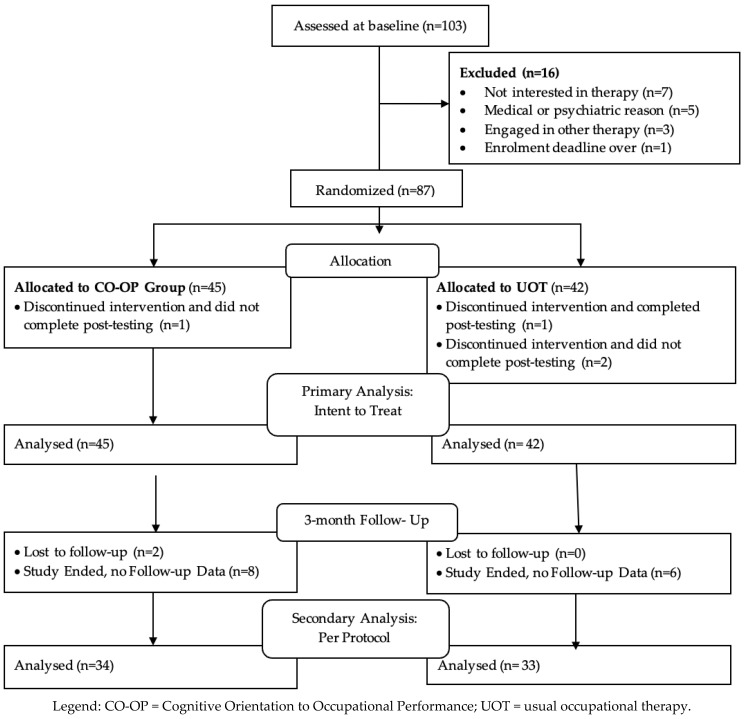
The CONSORT Flow Diagram.

Screening for eligibility was conducted with 252 individuals, of whom 149 were excluded (114 did not meet eligibility criteria, 24 were not interested, and 11 were unable to be reached). Participant flow through the study is depicted in the CONSORT diagram in Figure 1. Of 103 eligible participants assessed at baseline, fourteen were excluded for reasons indicated in the Figure, and 87 were randomized. Of these, four discontinued the intervention and withdrew from the study (three of these did not complete post-testing). Of the remaining 84, 2 were lost to follow-up following the post-test, and 14 were not tested at follow-up due to the study end. Thus, for intent-to-treat analyses, the sample size was 87. For per-protocol analysis at post-test, the sample size was 83, and it was 67 at follow-up.

Table 1 shows the socio-demographic characteristics or all participants. The two groups were similar at baseline, with the CO-OP group being slightly older (Mean difference 2.8 years, Cohen’s *d* = 0.21) and having slightly fewer years of education (Mean difference 0.8 years, Cohen’s *d* = −0.28). The proportion of participants reporting independence in self-care was high, although approximately 75% of the participants in both groups were living with some level of support and were retired, unemployed, and/or not seeking employment. Conversely, a very small proportion in both groups had full- or part-time paid employment. The CO-OP group was also rated as being slightly more engaged in their therapy (Mean difference 1.3 on Hopkins Rehabilitation Engagement Rating Scale, Cohen’s *d* = 0.25). As all calculated between-group effect size differences were negligible or small, and none of these variables were included as covariates in the planned ANCOVA.

### 3.1. Primary Outcome—COPM Untrained Goal Performance and Satisfaction with Goal Performance

The COPM standardized interview elicits goals related to everyday activities of daily living, including self-care, instrumental activities of daily living, socializing, productivity (work, school, and volunteer work), and leisure activities.

Table 2 shows the raw data for participants who completed post-testing (*n* = 84). The time effect for each of the scores was significant, with effect sizes being large (η^2^ = 0.19–0.59). As well, average change scores for three of the four COPM scores in the CO-OP group and two of the four in the UOT group were greater than two points, suggesting clinically important change was achieved.

As planned, ITT analysis was used for the four COPM scores at the primary endpoint (post-testing) using analyses of covariance. Among the four people who discontinued the intervention, three did not complete the post-tests and COPM, and so data for these three people were imputed. The ANCOVA model results are shown in Table 3. Pre-intervention scores were included in each model but were not found to be significant factors in any of the models. The analyses revealed that CO-OP training resulted in significantly higher COPM scores for both untrained and trained performance and satisfaction with performance (*p* ≤ 0.05).

Therapists in both study arms were allotted 15 sessions of therapy per participant. The decision to end therapy after fewer sessions was based on the therapist and participant agreeing that they had progressed as far as possible on the treatment goals. This decision was made more frequently in the UOT group, with participants in this group receiving fewer sessions (Mean ± SD; 8.1 ± 2.5) than those in the CO-OP group (Mean ± SD; 14.5 ± 2.0) (Cohen’s *d* = 2.85).

### 3.2. Secondary Outcomes

Secondary outcomes include COPM scores for both untrained and trained goals (performance and satisfaction) at follow-up, and psychosocial and neuropsychological scores at post-intervention and follow-up.

#### 3.2.1. COPM at Follow-Up

Raw scores for the 67 (CO-OP *n* = 34; UOT *n* = 33) participants who completed the three-month follow-up testing are shown in Table 4. At this point, between-group changes in trained goal performance and satisfaction from pre-test remained significantly greater (*p* ≤ 0.05) in the CO-OP group, with moderate effect sizes η^2^ = 0.06 to 0.11) (see Table 4). Between-group changes for untrained performance and satisfaction scores did not reach significance, although average changes in untrained goal satisfaction in the CO-OP group were greater than two points, suggesting clinically important change was maintained at 3 months.

#### 3.2.2. Executive Function and Community Integration Outcomes

Table 5 shows scores for the executive function and community integration outcomes at each time and the results of repeated measures ANOVA models comparing post-test and follow-up scores to pre-test scores. Between-group analyses showed no significant benefit for CO-OP over UOT for any of these measures, and effect sizes were negligible to small apart from the follow-up scores for the self-ordered pointing task. A significant effect was seen in favour of the CO-OP group, with a moderate effect size (η^2^ = 0.08). Within-group analyses showed that both groups of participants improved over time on the DKEFS-Trails 4 at follow-up, on the BRIEF-BRI at post-test, and on the participation score of the MPAI at post-test. Effect sizes were small to moderate for these changes.

## 4. Discussion

This large randomized controlled trial showed CO-OP therapy to be more effective than usual occupational therapy (UOT) at post-intervention for all four subscores of the Canadian Occupational Performance Measure (COPM) and at three-month follow-up for two subscores of the COPM. It appeared that both interventions were highly acceptable to participants, as only a few participants discontinued their allocated intervention, and engagement with rehabilitation in both groups was equitable. While participants in both groups experienced improvements of a magnitude to be considered clinically important [51], the results at post-intervention suggest that the CO-OP intervention was superior for achieving statistically significant positive benefits in the performance of, and satisfaction with, the meaningful everyday life activities addressed in the therapeutic process, and this extended to the performance of, and satisfaction with, activities that had not been addressed in therapy. This suggests that the learning achieved through the CO-OP process can be generalized and transferred to other activities in people’s everyday lives. The results of this study align with those from other randomized controlled trials of CO-OP in the stroke population [61], including four in which the control group received UOT [32,47,62,63]. Our study differs from these previous studies in two important ways: it was fully powered, and it included people with chronic ABI (stroke and traumatic brain injury). Results at the secondary time point showed some attenuation of these benefits and are discussed further below.

The transfer of learning that appears to have been achieved at post-intervention, particularly in the CO-OP group, implies that participants were able to take knowledge and/or skills learned while being trained for one activity and apply this learning to another activity. Training for generalization and transfer is an essential aspect of the CO-OP intervention, which is built into many of the essential elements and has been reported in many studies [25,42]. In this study, we adapted the intervention by being highly explicit about generalization and having participants practice using the meta-cognitive approach on other goals that had not been identified as training or untrained goals in their initial interviews. We hypothesize that this formal generalization and transfer work did not occur to the same extent, if at all, in UOT.

In the CO-OP arm, participants were also encouraged to develop a post-intervention follow-up plan for support and accountability with continuing their work. It is possible that these sessions accounted, in part, for the larger number of therapy sessions in the CO-OP arm. We posit that the development of this plan may have contributed to our finding that at three-month follow-up, we found CO-OP to be superior to UOT for achieving statistically significant changes in performance and satisfaction with trained goals. The magnitude of change for these scores, as well as for satisfaction with performance for untrained goals, was greater than two, suggesting these changes made a clinically significant difference [51]. Nevertheless, we found limited evidence in support of transfer of learning at three months.

We hypothesize that the other essential elements of CO-OP also contributed to the benefits we found. Therapists’ use of guided discovery, together with engaging participants in their own performance analysis, facilitates participants’ consideration of the plans they need to make to move them towards goal achievement and enables them to change plans without difficulty, as it is the plan that is understood to be the problem or solution rather than their own abilities or lack thereof. This process, we believe, also contributes to participants in the CO-OP process reporting consistent improvements in satisfaction with their performance, as we say in this study.

It is possible that the benefits we found were mitigated by a study design factor. All participants in this study set goals prior to starting the study, the majority of which were focused on everyday activities and participation in everyday life. While goal-setting is fundamental to client-centred rehabilitation, it is a practice that has not yet been routinely adopted [64,65]. The goal setting that occurred as part of the baseline outcome assessment may have facilitated goals being accomplished by all participants, as this process made their goals explicit to themselves and to their treating therapists. The importance of goal-setting in the rehabilitation context is well known. Involving clients in this process inherently respects their values and preferences and results in enhanced achievement of personally meaningful outcomes [66,67]. Goal-setting with clients with ABI is particularly important, as executive dysfunction, endemic in this population, interferes with successful goal-setting.

Our investigation of the secondary outcomes of improved community integration and improved executive function saw no differences between the treatment groups, except for a significant benefit in the self-ordered pointing test score at follow-up (relative to baseline) in favour of the CO-OP group. Self-ordered pointing (SOP) is a task associated with the processes of aspects of executive functioning, including strategy utilization, planning, and working memory [52]. To our knowledge, no other study has reported benefits on the SOP task related to the CO-OP intervention. However, benefits have been reported on other neuropsychological tests of executive processes, including the DKEFS Colour-Word Interference Task [29] and the DKEFS Trail Making Test Condition 4 [32], both tests of cognitive flexibility. As these two studies only included participants with acute or sub-acute ischemic stroke (within three months of stroke onset), improvement in executive function processes was anticipated. At the chronic state, this is less likely, but we are intrigued by our findings, since SOP benefits from the application of strategic processes. It is also possible that this finding is spurious. We hope that future studies of CO-OP in adults with ABI will consider including the SOP as part of their testing protocol.

We were encouraged to find that both CO-OP and UOT resulted in improvements over time on tests of executive function, including BRIEF-BRI. Having participants focus explicitly on meaningful everyday activities in their therapy may have resulted in improved executive functioning. However, it is possible that the increased familiarity of participants with the test procedure may have resulted in the improvements noted, although we used alternate versions of tests at each time point where available (e.g., DKEFS Condition 4) to minimize practice effects. We also note that the magnitude of change on the BRIEF-BRI was very small, given that the scale ranges from 0 to 120.

We hypothesize that the lack of change in overall community integration, as measured by the MPAI, can be attributed to the chronic nature of our sample and that all participants were living in the community. In our pilot work, we observed a significant benefit on the MPAI for community-dwelling adults with TBI receiving CO-OP, but this was relative to a wait-list control group [6]. Others have observed changes on the MPAI in chronic TBI samples, although in response to longer and more intensive interventions [56,68]. The CO-OP intervention is quite short, consisting, in this study, of approximately 15 h of 1:1 training over a two- to three-month period. It is of note, in this study, that relatively low doses of UOT, multiple years since the event, also resulted in clinically important changes in performance and satisfaction with performance of everyday meaningful activities. Future research should investigate the benefits of this type of training over longer time periods.

Meta-cognitive strategy training is recommended as a practice guideline for adults with traumatic brain injury (TBI) [17,69] and for adults with stroke [69] who have executive function deficits. Jeffay and colleagues [17] further state that this type of training should be conducted at least six months post-TBI and in context within the community. Cicerone and colleagues [69] state that this type of training should be incorporated into treatment for practical goals and applied in everyday situations. This study provides further evidence for the benefits of this type of training. We have also found that incorporating this type of training is beneficial for older adults with subjective cognitive impairment [70], to improve performance and satisfaction with performance of everyday life activities, to enhance the uptake of a brain-healthy diet for people with vascular risk factors, subjective cognitive impairment, and mild cognitive impairment [36], and to enhance the achievement of a minimally clinically important difference in the Functional Independence Measure following stroke inpatient rehabilitation when used by a stroke rehabilitation team [71]. Thus, we encourage all therapists working with ABI clients with executive dysfunction to consider incorporating CO-OP and/or other forms of meta-cognitive strategy training into their treatment plans that focus on changes in everyday activities.

## 5. Limitations

Despite extensive recruitment efforts, we failed to achieve the sample size of 90 (*n* = 45 per arm), which would have represented a fully powered trial. We were three people short. Nevertheless, to our knowledge, this is the largest trial of CO-OP in adults with ABI that has been conducted to date. Unfortunately, our grant funding did not permit us to complete three-month follow-up testing for 14 individuals. While a 3-month follow-up was our secondary outcome, we would have liked to conduct the more rigorous intent-to-treat analyses at this time point, but imputing scores for this number of individuals seemed imprudent. A third limitation is that the dose of therapy varied in the two groups. We were surprised that therapists and participants in the UOT arm agreed to end therapy, often well before the allowed 15 h were used. It is possible that if therapists delivering UOT had continued providing intervention, participants in this group would have seen further benefit. However, since therapy ended by mutual agreement between the therapist and participant, it is not clear what would have been provided in additional treatment sessions. We suggest that future research explore reasons why therapy has been discontinued, provide a stronger directive regarding the number of hours to be provided in both study arms, and record reasons for therapy being discontinued. Fourthly, our primary outcome was a self-report measure. It is possible that an objective measure of performance would show some discrepancies with self-report. While the COPM is a widely used, standardized interview, it would be ideal to include objective performance measures in future research. Finally, as we did not collect data on gender or ethnicity, it is possible that these factors may have influenced outcomes.

## 6. Conclusions

This large trial, comparing CO-OP to usual occupational therapy, found CO-OP to be significantly more effective at post-intervention for self-reported performance and satisfaction with performance of meaningful everyday life activities that had been trained in therapy and that had not been trained in therapy, suggesting that transfer of learning is achieved through this approach.

## Figures and Tables

**Table 1:** Baseline characteristics of participants (*n* = 87).

	CO-OP (*n* = 45)	Usual OT (*n* = 42)	Effect Size *
Sex, male, *n* (%)	26 (57.8)	29 (69)	0.11 *
Age, years, M (SD)	57.5 (13.9)	54.7 (13.3)	0.21
Education, years, M, SD	15.2 (2.9)	16.0 (3.2)	−0.28
Time since event, years, M, SD	5.3 (6.5)	6.2 (7.7)	−0.07
Pre-morbid IQ, Shipley, M, SD	28.8 (8.0)	29.0 (5.9)	−0.03
Proportion with TBI **^†^**	11 (24.4)	8 (19.0)	0.07 *
Executive Dysfunction, Adapted Exit-25, M, SD **^‡^**	2.7 (1.7)	2.6 (1.7)	0.01
Depression, PHQ-9, M, SD **^§^**	5.6 (4.04)	5.6 (4.73)	0.06
Proportion Living with support, *n* (%)	34 (75.6)	31 (73.8)	0.06 *
Proportion Independent in Self-Care	29 (64.4)	31 (73.8)	0.01 *
Proportion requiring 25% or more assistance with self-care	10 (22.2)	5 (11.9%)	0.01 *
Proportion with Full or Part-Time Paid Employment	6 (13.3)	3 (7.2)	0.01 *
Proportion Retired, Unemployed, Not seeking employment	35 (77.8)	34 (78.5)	0.01 *
Participant Engagement in therapy (** HRESR) M, SD	26.1 (3.0)	24.8 (4.3)	0.25

Legend: * Effect size Cohen’s *w* (otherwise, Cohen’s *d*); M = mean; SD = standard deviation; ^†^ TBI = traumatic brain injury, and non-TBI diagnoses included stroke, aneurysm, non-malignant brain tumour, and anoxia; **^‡^** Exit-25 adapted for telephone use; **^§^** PHQ-9 = Patient Health Questionnaire-9; ** HRERS = Hopkins Rehabilitation Engagement Rating Scale.

**Table 2:** COPM scores at baseline and post-intervention (*n* = 84).

COPM Scores	Group	Baseline (T1) Mean (SD)	Post-Intervention (T2) Mean (SD)	T2–T1, Change Score, Mean (SD)	Time Effect Size (η^2^)
**Performance-untrained**	CO-OP	4.00 (2.00)	5.36 (2.03)	1.36 (2.77)	0.19 ***
	UOT	3.04 (1.55)	4.29 (2.35)	1.25 (2.73)	
Performance-trained	CO-OP	3.66 (1.66)	6.65 (1.87)	**2.99 (2.49)**	0.54 ***
	UOT	3.02 (1.59)	5.34 (2.15)	**2.32 (2.38)**	
**Satisfaction-untrained**	CO-OP	3.23 (1.77)	5.36 (2.46)	**2.13 (2.71)**	0.25 ***
	UOT	2.92 (1.85)	3.99 (2.36)	1.07(2.96)	
Satisfaction-trained	CO-OP	2.88 (1.77)	6.59 (2.14)	**3.71 (2.67)**	0.59 ***
	UOT	2.70 (1.47)	5.14 (2.37)	**2.44 (2.35)**	

Legend: CO-OP = Cognitive Orientation to daily Occupational Performance Intervention; UOT = Usual Occupational Therapy. COPM = Canadian Occupational Performance Measure; bolded score names indicate scores for the primary outcome; bolded cells indicate post-intervention change that was greater than 2.0 points, suggesting a clinically important difference. *** *p* ≤ 0.001.

**Table 3:** Primary analysis: Intent to treat COPM at post-intervention.

	Tx Coefficient Estimate	SE	z-Value	*p*-Value
**Performance-untrained**	−0.99	0.49	−1.99	0.05
Performance-trained	−1.24	0.45	−2.75	0.007
**Satisfaction-untrained**	−1.29	0.52	−2.45	0.02
Satisfaction-trained	−1.43	0.51	−2.82	0.006

Legend: COPM = Canadian Occupational Performance Measure. Bolded score names indicate scores for the primary outcome.

**Table 4:** Secondary analysis: Pre-protocol COPM Scores (*n* = 67).

	Group	Baseline (T1) Mean (SD)	3-Month Follow-Up (T3), Mean (SD)	Change Score, T3–T1, Mean (SD)	Time Effect Size (η^2^)	Time x Group Interaction Effect Size (η^2^)
**Performance-untrained**	CO-OP	4.12 (1.88)	5.56 (2.28)	1.44 (2.57)	0.30 ***	0.06 *
	UOT	3.12 (1.57)	5.02 (2.40	1.90 (2.55)		
Performance-trained	CO-OP	3.70 (1.68)	6.32 (1.81)	**2.62 (2.21)**	0.48 ***	0.11 **
	UOT	3.23 (1.54)	4.98 (2.01)	1.75 (2.41)		
**Satisfaction-untrained**	CO-OP	3.31 (1.60)	5.49 (2.50)	**2.18 (2.82)**	0.33 ***	0.04 *
	UOT	2.84 (1.96)	4.53 (2.53)	1.67 (2.73)		
Satisfaction-trained	CO-OP	2.91 (1.72)	6.12 (2.30)	**3.21 (2.62)**	0.49 ***	0.06 **
	UOT	2.83 (1.47)	4.67 (2.45)	1.83 (2.61)		

Legend: CO-OP = Cognitive Orientation to daily Occupational Performance Intervention; UOT = Usual Occupational Therapy. COPM = Canadian Occupational Performance Measure; η^2^ = Partial eta squared. Bolded cells indicate follow-up intervention change that is greater than 2.0 points, suggesting a clinically important difference. * 0.1 > *p* > 0.05; ** *p* ≤ 0.05; *** *p* ≤ 0.001.

**Table 5:** Secondary analysis: Pre-protocol COPM executive function and community integration scores.

	Time Point	CO-OP	UOT	Time Effect Size (η^2^)	Between Group Effect Size (η^2^)
Executive Function Outcomes					
Self-Ordered Pointing	Baseline	3.95 (2.25)	4.26 (2.19)		
	Post	3.84 (2.60)	4.36 (2.21)	0.00	0.01
	Follow-up	3.38 (1.83)	4.94 (4.56)	0.00	0.08 **
DKEFS-Trails 4	Baseline	152.45 (61.89)	173.76 (71.44)		
(time in seconds)	Post	147.61 (68.02)	163.87 (75.16)	0.07 *	0.02
	Follow-up	144.71 (69.18)	166.50 (70.94)	0.11 **	0.11
DKEFS-Letter Fluency	Baseline	26.84 (11.92)	28.13 (13.22)		
(total correct)	Post	27.36 (12.98)	28.00 (13.51)	0.00	0.00
	Follow-up	28.27 (12.84)	27.93 (15.28)	0.02	0.00
BRIEF-BRI	Baseline	48.77 (10.91)	48.49 (11.21)		
	Post	45.88 (9.86)	47.03 (11.86)	0.08 **	0.00
	Follow-up	48.00 (10.97)	47.48 (12.78)	0.04 *	0.00
BRIEF-MI	Baseline	69.50 (15.25)	64.67 (15.17)		
	Post	65.52 (13.82)	64.77 (16.37)	0.05 *	0.01
	Follow-up	67.21 (14.30)	65.06 (17.30)	0.02	0.01
Community Integration Outcomes					
MPAI-Ability	Baseline	14.25 (7.89)	11.79 (6.69)		
	Post	13.77 (8.10)	11.90 (8.24)	0.00	0.02
	Follow-up	12.47 (6.74)	12.42 (9.30)	0.01	0.00
MPAI-Participation	Baseline	11.66 (5.35)	10.28 (4.39)		
	Post	10.51 (5.43)	9.62 (4.89)	0.04 *	0.02
	Follow-up	10.59 (4.88)	11.25 (6.03)	0.00	0.00

Legend: For all measures, lower scores represent better performance. DKEFS = Delis–Kaplan Executive Function System; BRIEF = Behaviour Rating of Executive Function; GEC = Global Executive Composite; BRI = Behavioural Regulation Index; MI = Metacognition Index; * 0.1 > *p* > 0.05; ** *p* ≤ 0.05.

## Data Availability

The data are not publicly available as this has not been approved by the Baycrest REB. Please contact the primary author for data access.
